# Feeding Patterns and Predictors of Malnutrition in Infants from Poor Socioeconomic Areas in Pakistan: A Cross-sectional Survey

**DOI:** 10.7759/cureus.452

**Published:** 2016-01-07

**Authors:** Muhammad Umer Nisar, Malik Muhammad Anwar ul Haq, Saad Tariq, Madiha Anwar, Anam Khawar, Ahmed Waqas, Anam Nisar

**Affiliations:** 1 Medical Student, Yusra Medical and Dental College, Islamabad, Pakistan; 2 District Headquarter Hospital, Sargodha, Pakistan; 3 Dow Medical College, Karachi, Pakistan; 4 Combined Military Hospital, Mangla, Pakistan; 5 Final year MBBS Student, CMH Lahore Medical College and Institute of Dentistry, Lahore Cantt, Pakistan; 6 POF Hospital, Wah Cantt, Pakistan

**Keywords:** malnutrition, children, breast feeding, nutrition

## Abstract

Introduction: Malnutrition, a state of under or over nutrition caused by improper food intake, causes significant morbidity and mortality in developing countries. It leads to a number of diseases which can be further divided into those caused by protein-caloric malnutrition and those caused by vitamin deficiencies, micronutrient, and mineral deficiencies. The purpose of this study was to identify the factors which contribute to malnutrition and to assess the dietary pattern in the pediatric population from birth up to five months belonging to poor socioeconomic areas. The children in this sample presented to a tertiary care hospital in the district of Sargodha, Pakistan. The findings in this cohort will support the development of an effective plan to tackle these issues.

Methods: This cross-sectional study was undertaken between June 2014 and December 2014 at the inpatient pediatric department of District Headquarter Hospital Sargodha. Data were collected and recorded on a predesigned form which consisted of four sections to record 1) demographics: parents' level of education, monthly income, number of dependent household members, and birth interval; 2) anthropometric and personal history, birth history, and degree of malnutrition; 3) any secondary causes of malnutrition; and 4) feeding history. The data were analyzed in SPSS v. 20. Chi-squared, phi statistics, and logistic regression analysis were run to analyze the data.

Results: A total of 294 participants were included in the study. Logistic regression analysis showed that the degree of malnutrition was associated negatively with increasing age and positively with family size. A majority of children (144, 49%) were being breastfed for less than 5 minutes followed by 38 (13%) > 5 minutes to 15 minutes, and 2 (0.7%) > 15 minutes while 110 (37 %) infants were not breastfed. Children who were breastfed were less likely to have severe malnutrition than those who were given formula, fresh cow's or goat's milk, or more than one type of food.

Conclusion: Children who were breastfed were less likely to have severe malnutrition. The degree of malnutrition was negatively associated with age and positively associated with family size.

## Introduction

South Asian countries have the highest burden of malnourished children under five years of age [1-3]. Half of the children in South Asia are malnourished, and half of world’s malnourished children live in Pakistan, Bangladesh, and India [3-4]. Malnutrition has multifactorial effects on physical, social, mental, and intellectual development, which highlights the importance of proper nutrition in children [5].

Pakistan has the highest child and infant mortality rate in South Asia [6]. The mortality in children under five years of age was 101 per 1000 live births, as reported in 2005, with malnutrition being a major contributor [4, 7]. A national survey in Pakistan showed that one-third of the children are malnourished, 6.2-8.3 million children (about 30%-40%) have low height for their age (stunting), and more than 2.9 million children (14%) have low weight for their height (wasting) [8]. Another national survey in this country also showed that during the decade from 1990 to 2001, the proportion of underweight children under five years of age had decreased slightly from 40.1% to 37.4%; however, the prevalence of wasting had increased from 11.8% to 14.9%, and the prevalence of stunting had increased from 36.3% to 40% [4]. This indicates that malnutrition is a developing problem for children in Pakistan – an issue that poses a threat to the achievement of the United Nations Millennium Development Goal number 4, i.e. to reduce child mortality by 2015. To highlight the seriousness of this issue, this report presents the key determinants of malnutrition among young Pakistani children.

The present study was designed to determine the feeding patterns and predictors of malnutrition in children from birth up to five months of age in Pakistan.

## Materials and methods

This cross-sectional study was undertaken between June 2014 and December 2014 in the District Headquarter Hospital (DHQ) Sargodha, Pakistan. Ethical approval was obtained from the Ethical Review Committee of Yusra Medical and Dental College, Islamabad. Two hundred and ninety-four children under five months of age followed at the inpatient department of DHQ Sargodha were included in the study. The minimum sample size required for the survey was calculated to be 384 based on a 95% confidence level, 5% margin of error, and a population of 11.2 million children suffering from malnutrition in Pakistan [8-9]. However, due to limited resources, we could not achieve the required sample size. Therefore, the results from this study are not generalizable to the Pakistani populace.

Written consent was obtained from all participants following a comprehensive explanation of the purposes of the study. Children with serious illness or comorbidities were excluded from the study. Detailed history and anthropometric measurements were taken by a physician.

Data were collected and recorded on a pretested, predesigned form which consisted of four sections. The first section recorded demographics, parents’ level of education, monthly income, the number of dependent household members, and birth interval. The second section recorded anthropometric data and personal history, birth history, and degree of malnutrition. The degree of malnutrition were categorized as first degree (75-90% of expected weight), second degree (60-75% of expected weight), and third degree (<60% of expected weight). The third section recorded any secondary cause(s) of malnutrition. The fourth section recorded feeding history, including details about breastfeeding, formula feeding, and fresh cow’s or goat’s milk feeding. 

The data were analyzed in SPSS v. 21 (IBM, Chicago, Illinois). Frequencies, percentages, and the chi-squared goodness of fit test were run for participants’ characteristics and feeding patterns. Logistic regression analysis was used to elucidate the predictors of severity of malnutrition in the infants. Covariates entered into the logistic regression model were the age of the infant, gender, mother’s education, income, family size, background, gestation, mode of delivery, and type of feeding. The degree of malnutrition was dichotomized as “first degree” and “severe” (second, plus third-degree malnutrition) and entered as a dependent variable.

## Results

The mean age of the infants was 3.51 months and the mean number of family members was 7.14. Most of the infants included in the survey were males, had illiterate mothers, had family income < Rs. 20,000/month (200 USD) and belonged to rural areas. Most of the respondents had SVD and partial immunization status. Almost 45% of the infants were being breastfed. Detailed results are given in Table 1.

**Table 1:** Characteristics of the Children Surveyed (n = 294)

Characteristics		Frequency (n)	Percentage (%)	Chi Square P-value
Gender	Male	173	58.80%	< .001
	Female	121	42.20%	
Mothers' Education	Cannot read and write	195	66.30%	< .001
	Primary School	41	13.90%	
	Secondary School	49	16.70%	
	Higher Secondary School	9	3%	
Monthly Income (combined)	< 95 USD	101	34.50%	< .001
	95-190 USD	181	61.80%	
	> 190 USD	11	3.80%	
Background	Urban	117	39.80%	< .001
	Rural	177	60.20%	
Gestation	Term	290	98.60%	< .001
	Preterm	4	1.40%	
Feeding	Mother feed	132	44.90%	< .001
	Formula feed	36	12.20%	
	Fresh feed	74	25.20%	
	More than one type	52	17.70%	
Mode of Delivery	SVD	217	73.80%	
	SVD with forceps/vacuum/episiotomy	17	5.80%	< .001
	Cesarean section	60	20.40%	
Immunization Status	Up to date	115	39.10%	< .001
	partial	121	41.20%	
	none	58	19.70%	
Degree of Malnutrition	1st Degree	70	23.80%	< .001
	2nd Degree	96	32.70%	
	3rd Degree	128	43.50%	

Table 2 presents the feeding patterns of the infants feeding on mothers’ breast milk. Only 184 (62.59%) infants were receiving mother’s milk either alone or combined with other food. Most of the infants were being fed mother’s milk < 5 times/day, 2 times/ night and for < 5 minutes duration/session. Most commonly cited reason for not breastfeeding was personal preference of the mother and nonavailability/less amount despite spontaneous vaginal delivery (SVD).

**Table 2:** Breast feeding patterns in malnourished infants (n=184*) * Includes infants in “more than one type of food” category taking breast milk along with other food type.

Variable		Frequency (n)	Percentage (%)	Chi Square P- value
No. of Mother Feeds Given/Day	< 5	148	80.4%	< .001
	5 to 10	36	19.6%	
	> 10	0	0.0%	
No. of Mother Feeds Given/Night	0	8	4.4%	< .001
	1	67	36.4%	
	2	93	50.5%	
	3	13	7.1%	
	4	3	1.6%	
Duration/Feed of Mothers	< 5 min	144	78.3%	< .001
	5-15 min	38	20.7%	
	> 15 min	2	1.0%	
Reason of No Mother Feed	Mother on drugs	0	0.0%	< .001
	Twin, triplets or quadruplets	5	2.7%	
	Mothers' Illness (organic or psychiatric)	8	4.4%	
	Hot/cold food concept	1	0.5%	
	Mothers' feed not good for baby	5	2.7%	
	Mother is malnourished	3	1.6%	
	Due to C-Section	13	7.1%	
	Personal preference	57	31.0%	
	Non-availability/less amount despite SVD	34	18.5%	
	No response	58	31.5%	

Less than 40% of the infants were receiving fresh food. Most of them were receiving cow’s milk, < 5 times/day and 2 times/night. Most of the infants (104/109) received diluted fresh food and the most cited reason for over-dilution was family pressures/financial constraints. Table 3 details the feeding patterns of infants feeding on fresh food.

**Table 3:** Pattern of Feeding of Fresh Food to the Infant (n= 109*) * Includes infants in “more than one type of feed” category taking fresh feed along with other food type. ^1 ^Contains one to five missing values

Variable		Frequency (n)	Percentage (%)	Chi Square P-value
Type of Fresh Feed	Cows’ milk	107	98.2%	< .001
	any other	2	1.8%	
No. of Fresh Feeds Given/Day	< 5	100	91.7%	< .001
	5 to 10	9	8.3%	
	> 10	0	0.0%	
No. of Fresh Feeds Given/Night^1^	0	5	4.6%	< .001
	1	37	33.9%	
	2	51	46.8%	
	3	15	13.8%	
Fresh Feed Over-dilution Reason^1^	No awareness	24	22.0%	< .001
	Perceived intolerance to undiluted milk	8	7.3%	
	Family pressures/financial constraints	67	61.2%	
	No over-dilution	5	4.6%	

Many of the infants (56, 19%) were receiving formula food, < 5 times/day and 2 times/night. Nine (9/56) infants were receiving over-diluted food and the most commonly cited reason for over-dilution was no awareness and family pressures/financial constraints. Table 4 details the feeding patterns of infants feeding on formula food.

**Table 4:** Pattern of Formula Feed of the Infants Surveyed (n=56*) * Includes infants in “more than one type of feed” category taking fresh feed along with other food type.

Variable		Frequency (n)	Percentage (%)	Chi square P-value
No. of Formula Feeds Given/Day	< 5	47	83.9%	< .001
	5 to 10	9	16.1%	
	> 10	0	0.0%	
No. of Formula Feeds Given/Night	0	2	3.6%	< .001
	1	19	33.9%	
	2	30	53.6%	
	3	5	8.9%	
Dilution/Feed of Formula	1 spoon/ounce	41	73.2%	< .001
	1/2 spoon/ounce	9	16.1%	
	1/3 spoon/ounce	6	10.7%	
Formula Feed Over-dilution Reason	No awareness	9	16.1%	< .001
	Perceived intolerance to undiluted milk	0	0.0%	
	Family pressures/financial constraints	5	8.9%	
	No over-dilution	42	75.0%	

According to logistic regression analysis, the degree of malnutrition was negatively associated with increasing age and positively with family size. Children who were taking mother’s food were less likely to have severe malnutrition than those who were having formula food, fresh food, or more than one type of food.

**Table 5:** Logistic Regression Analysis (Backwards Method) for Degree of Malnutrition in Children Presenting at the Hospital (n = 294) Model Chi square = 59.9, P < .001, Hosmer and Lemeshow, P = .95 Cox and Snell R square = .17, Negelkerke R Square = .26

Variables	Odds Ratio	P-value	95% C.I.for Odds Ratio	
			Lower	Upper
Age	0.505	0	0.392	0.65
Family size	1.123	0.039	1.006	1.253
More than one feed		0.004		
Mother feed	0.197	0.002	0.07	0.558
Formula feed	0.515	0.352	0.128	2.08
Fresh feed	0.462	0.173	0.152	1.402
Constant	60.923	0		

## Discussion

Malnutrition in children is a serious health hazard in a developing country like Pakistan. We searched for factors that contribute to malnutrition in children under five months of age from families with poor socioeconomic status. Our analysis showed that malnutrition was negatively associated with increasing age and positively associated with family size. We also found that children who were breastfed were less likely to have severe malnutrition.

Among the factors associated with malnutrition are the parents’ education, income, family size, the birth interval with the older sibling, and feeding pattern. Many studies have shown a significant association between malnutrition and the parents’ education, especially the mother’s. Ali, et al. reported a significant difference in malnutrition (as shown by low weight) between children whose mothers were illiterate compared to educated mothers in Karachi (P < 0.016) [10]. Islam, et al. showed that maternal illiteracy and lack of breastfeeding were associated with a four-fold increase in the risk of severe malnutrition in children [11]. Smith, et al., in their study of 63 developing countries for 25 years, concluded that the mother’s secondary school enrollment had a strong negative association with child malnutrition, once again highlighting the importance of maternal education [12]. Paternal education also has an impact on child malnutrition because the father is usually the sole earner and decision maker in the family in developing countries, such as Pakistan. Jesmin, et al. showed that a one-year increase in the father’s schooling was associated with an 11% reduction in stunting [13]. A study by Rayhan, et al. showed that the prevalence of underweight was lower in children whose fathers had a higher level of education [14]. Memon, et al. showed that feeding practices are mostly affected by the mother’s education (P < 0.0001) and income (P < 0.0003) [15].

Family income is also important for child nutrition. Less income is associated with inadequate nutrition, poor sanitation, and an increased risk of infection, which leads to stunting in children [16-18]. Kavosi, et al. showed that stunting was significantly associated with lower family income (OR 3.21, CI: 1.17 – 8.85), and children in families with an income higher than 667 US dollars were less likely to be stunted compared to those with an income of 334 US dollars or less [19]. In the present study, however, we found no significant association between the parents’ education or income and malnutrition.

Family size is also an important factor in malnutrition. A study done in India showed that the overall prevalence of protein-energy malnutrition was significantly higher (P < 0.001) in infants from joint families than those from nuclear families. In large families, 74.6% of children had malnutrition vs. only 21.96% in small families [20]. Our study also showed that family size was positively associated with malnutrition, according to the logistic regression analysis.

Regarding birth interval, a study of 1,884 children showed that children who had less than a 24-month birth interval with the older sibling had increased the risk of malnutrition – a result that points toward the potential benefits of longer birth intervals [21].

Breastfeeding is a cost-effective mechanism to reduce malnutrition in developing countries. The World Health Organization recommends that exclusive breastfeeding should be started soon after birth and continued for six months, followed by the addition of complementary food up to two years of age [22]. A previous study in Pakistan showed that about 27.2% of infants (95% CI: 26.0, 28.3) started breastfeeding within one hour of birth and 65.5% (95% CI: 64.3, 66.8) within one day [23]. A study in Nepal showed that about 7.7% of all neonatal deaths might be avoided if breastfeeding were started on the first day of life, and 19.1% of the deaths might be avoided if breastfeeding were started within the first hour after birth [24]. Another study in Pakistan showed that almost all infants are breastfed, but that inadequate feeding practices led to poor nutrition and child mortality [25]. Exclusive breastfeeding for the first six months is the single most important factor in decreasing child morbidity and mortality [26]. Improving feeding practices will contribute to the achievement of the United Nations Millennium Development Goals. Improvements can be achieved through public awareness and health education about the importance of breastfeeding and appropriate complementary feeding in children in accordance with the Integrated Management of Neonatal Child Illness (IMNCI) Feeding Assessment Guidelines for young infant feeding [27].

Pakistan is an underdeveloped country where most of the population lives in rural areas. Mothers in these areas often have a low level of education and live in poverty, and these factors have an especially marked influence on infant and child nutrition. In addition, people in rural areas have less access to basic health care facilities. There are also misconceptions about the foods given to children, according to traditional practices without an actual scientific basis. Because of the multifactorial origin of malnutrition, appropriate intervention to reduce its incidence and prevalence in Pakistan should include: 1) education of mothers about healthy feeding practices by the Lady Health Visitors (LHV) in rural areas, 2) public awareness through mass media and community participation, and 3) the development of programs by the government and non-government organizations to provide care to malnourished children. Although great efforts are being made by WHO and the government, continued work is still needed.

## Conclusions

Malnutrition in children is associated with poor breastfeeding practices. Children who were breastfed were less likely to suffer from malnutrition. More than one-third of the infants were not breastfed, mainly because of the personal preference of the mother and less amount of breast milk. These poor feeding practices can be improved by education of the mothers. The degree of malnutrition was negatively associated with age and positively associated with family size.
